# Supplementary material for: Transfer of training—Virtual reality training with augmented multisensory cues improves user experience during training and task performance in the real world
Source: PLoS One. 2021 Mar 24;16(3):e0248225. doi: 10.1371/journal.pone.0248225 (PMC7990292; doi:10.1371/journal.pone.0248225)
Supplement: S1 Questionnaire — (PDF) [file pone.0248225.s001.pdf]

IMMERSIVE TENDENCIES QUESTIONNAIRE

(Witmer & Singer, Version 3.01, September 1996)*
Revised by the UQO Cyberpsychology Lab (2004)

Name: _______________ Age:____ Sex: _____ Occupation__________________

Indicate your preferred answer by marking an "X" in the appropriate box of the seven point scale. Please consider the entire scale when making your responses, as the intermediate levels may apply. For example, if your response is once or twice, the second box from the left should be marked. If your response is many times but not extremely often, then the sixth (or second box from the right) should be marked.

1. Do you easily become deeply involved in movies or TV dramas?
|________|________|__________|_________|_________|_________|_________|
NEVER OCCASIONALLY OFTEN

2. Do you ever become so involved in a television program or book that people have problems getting your attention?

|________|________|__________|_________|_________|_________|_________| NEVER
OCCASIONALLY OFTEN

3. How mentally alert do you feel at the present time?

|________|________|__________|_________|_________|_________|_________|
NOT ALERT MODERATELY FULLY ALERT

4. Do you ever become so involved in a movie that you are not aware of things happening around you?

|________|________|__________|_________|_________|_________|_________| NEVER
OCCASIONALLY OFTEN

5. How frequently do you find yourself closely identifying with the characters in a story line?

|________|________|__________|_________|_________|_________|_________| NEVER
OCCASIONALLY OFTEN

6. Do you ever become so involved in a video game that it is as if you are inside the game rather than moving a joystick and watching the screen?

|__________|__________|_____________|___________|___________|___________|___________| NEVER

OCCASIONALLY OFTEN

7. How physically fit do you feel today?

|_________|_________|___________|__________|__________|__________|__________|

NOT FIT MODERATELY FIT EXTREMELY FIT

8. How good are you at blocking out external distractions when you are involved in something?

|_________|_________|___________|__________|__________|__________|__________|

NOT VERY GOOD SOMEWHAT GOOD VERY GOOD

9. When watching sports, do you ever become so involved in the game that you react as if you were one of the players?

|_________|_________|___________|__________|__________|__________|__________|

NEVER OCCASIONALLY OFTEN

10. Do you ever become so involved in a daydream that you are not aware of things happening around you?

|_________|_________|___________|__________|__________|__________|__________| NEVER

OCCASIONALLY OFTEN

11. Do you ever have dreams that are so real that you feel disoriented when you awake?

|_________|_________|___________|__________|__________|__________|__________| NEVER

OCCASIONALLY OFTEN

12. When playing sports, do you become so involved in the game that you lose track of time?

|_________|_________|___________|__________|__________|__________|__________|

NEVER OCCASIONALLY OFTEN

13. How well do you concentrate on enjoyable activities?

|_________|_________|___________|__________|__________|__________|__________| NOT

AT ALL MODERATELY VERY WELL

WELL

14. How often do you play arcade or video games? (OFTEN should be taken to mean every day or every two days, on average.)
|_________|_________|___________|__________|__________|__________|__________|
NEVER OCCASIONALLY OFTEN

15. Have you ever gotten excited during a chase or fight scene on TV or in the movies?

|_________|_________|___________|__________|__________|__________|__________|
NEVER OCCASIONALLY OFTEN

16. Have you ever gotten scared by something happening on a TV show or in a movie?

|_________|_________|___________|__________|__________|__________|__________|
NEVER OCCASIONALLY OFTEN

17. Have you ever remained apprehensive or fearful long after watching a scary movie?

|_________|_________|___________|__________|__________|__________|__________|
NEVER OCCASIONALLY OFTEN

18. Do you ever become so involved in doing something that you lose all track of time?

|_________|_________|___________|__________|__________|__________|__________|
NEVER OCCASIONALLY OFTEN
