# Supplementary material for: Transfer of training—Virtual reality training with augmented multisensory cues improves user experience during training and task performance in the real world
Source: PLoS One. 2021 Mar 24;16(3):e0248225. doi: 10.1371/journal.pone.0248225 (PMC7990292; doi:10.1371/journal.pone.0248225)
Supplement: S2 Questionnaire — (PDF) [file pone.0248225.s002.pdf]

PRESENCE QUESTIONNAIRE

(Witmer & Singer, 2005)
Revised by the UQO Cyberpsychology Lab (2004)

Name:_____________ Age:_____ Sex: _____ Occupation:_________________

Characterize your experience in the environment, by marking an "X" in the appropriate box. Please consider the entire scale when making your responses, as the intermediate levels may apply. Answer the questions independently in the order that they appear. Do not skip questions or return to a previous question to change your answer.

WITH REGARD TO THE EXPERIENCED ENVIRONMENT

1. How much were you able to control events?

|________|________|__________|_________|_________|_________|_________| NOT AT ALL SOMEWHAT COMPLETELY

2. How responsive was the environment to actions that you initiated (or performed)?

|________|________|__________|_________|_________|_________|_________|
NOT RESPONSIVE MODERATELY RESPONSIVE COMPLETELY RESPONSIVE

3. How natural did your interactions with the environment seem?

|________|________|__________|_________|_________|_________|_________|
EXTREMELY ARTIFICIAL BORDERLINE COMPLETELY NATURAL

4. How much did the visual aspects of the environment involve you?

|________|________|__________|_________|_________|_________|_________| NOT AT ALL SOMEWHAT COMPLETELY

5. How much did the auditory aspects of the environment involve you?

|________|________|__________|_________|_________|_________|_________| NOT AT ALL SOMEWHAT COMPLETELY

6. How natural was the mechanism which controlled movement through the environment?

|________|________|__________|_________|_________|_________|_________|
EXTREMELY ARTIFICIAL BORDERLINE COMPLETELY NATURAL

7. How compelling was your sense of objects moving through space?

|________|________|__________|_________|_________|_________|_________|
NOT CONSISTENT MODERETELY CONSISTENT VERY CONSISTENT

8. How much did your experiences in the virtual environment seem consistent with your real world experiences?

|________|________|__________|_________|_________|_________|_________|
NOT CONSISTENT MODERATELY CONSISTENT VERY CONSISTENT

9. Were you able to anticipate what would happen next in response to the actions that you performed?

|________|________|__________|_________|_________|_________|_________|
NOT AT ALL SOMEWHAT COMPLETELY

10. How completely were you able to actively survey or search the environment using vision?

|________|________|__________|_________|_________|_________|_________| NOT AT ALL SOMEWHAT COMPLETELY

11. How well could you identify sounds?

|________|________|__________|_________|_________|_________|_________| NOT AT ALL PRETTY CLOSELY VERY CLOSELY

12. How well could you localize sounds?

|________|________|__________|_________|_________|_________|_________| NOT AT ALL PRETTY CLOSELY VERY CLOSELY

13. How well could you actively survey or search the virtual environment using touch?

|________|________|__________|_________|_________|_________|_________| NOT AT ALL PRETTY VERY

CLOSELY CLOSELY

14. How compelling was your sense of moving around inside the virtual environment?

|________|________|__________|_________|_________|_________|_________| NOT

MODERATELY VERY
COMPELLING COMPELLING COMPELLING

15. How closely were you able to examine objects?

|________|________|__________|_________|_________|_________|_________|
NOT AT ALL PRETTY VERY
CLOSELY CLOSELY

16. How well could you examine objects from multiple viewpoints?

|________|________|__________|_________|_________|_________|_________| NOT
AT ALL SOMEWHAT EXTENSIVELY

17. How well could you move or manipulate objects in the virtual environment?
|________|________|__________|_________|_________|_________|_________| NOT
AT ALL SOMEWHAT EXTENSIVELY

18. How involved were you in the virtual environment experience?
|________|________|__________|_________|_________|_________|_________|
NOT MILDLY COMPLETELY
INVOLVED INVOLVED ENGROSSED

19. How much delay did you experience between your actions and expected outcomes?
|________|________|__________|_________|_________|_________|_________|
NO DELAYS MODERATE LONG
DELAYS DELAYS

20. How quickly did you adjust to the virtual environment experience?
|________|________|__________|_________|_________|_________|_________| NOT
AT ALL SLOWLY LESS THAN
ONE MINUTE

21. How proficient in moving and interacting with the virtual environment did you feel at the end of the experience?
|________|________|__________|_________|_________|_________|_________| NOT
REASONABLY VERY
PROFICIENT PROFICIENT PROFICIENT

22. How much did the visual display quality interfere or distract you from performing assigned tasks or required activities?
|________|________|__________|_________|_________|_________|_________| NOT
AT ALL INTERFERED PREVENTED
SOMEWHAT TASK PERFORMANCE

23. How much did the control devices interfere with the performance of assigned tasks or with other activities?
|________|________|__________|_________|_________|_________|_________|
NOT AT ALL INTERFERED INTERFERED
SOMEWHAT GREATLY

24. How well could you concentrate on the assigned tasks or required activities rather than on the mechanisms used to perform those tasks or activities?
|________|________|__________|_________|_________|_________|_________|
NOT AT ALL SOMEWHAT COMPLETELY

25. How completely were your senses engaged in this experience?
|________|________|__________|_________|_________|_________|_________|
NOT AT ALL SOMEWHAT COMPLETELY

26. To what extent did events occurring outside the virtual environment distract from your experience in the virtual environment?
|________|________|__________|_________|_________|_________|_________|
NOT AT ALL SOMEWHAT COMPLETELY
DISTRACTED DISTRACTED

27. Overall, how much did you focus on using the display and control devices instead of the virtual experience and experimental tasks?
|________|________|__________|_________|_________|_________|_________|
NOT AT ALL SOMEWHAT COMPLETELY

28. Were you involved in the experimental task to the extent that you lost track of time?
|________|________|__________|_________|_________|_________|_________|
NOT MILDLY COMPLETELY
INVOLVED INVOLVED ENGROSSED

29. How easy was it to identify objects through physical interaction, like touching an object, walking over a surface, or bumping into a wall or object?
|________|________|__________|_________|_________|_________|_________| NOT
AT ALL SOMEWHAT COMPLETELY

30. Were there moments during the virtual environment experience when you felt completely focused on the task or environment?

|________|________|__________|_________|_________|_________|_________| NOT AT ALL SOMEWHAT COMPLETELY

31. How easily did you adjust to the control devices used to interact with the virtual environment?

|________|________|__________|_________|_________|_________|_________|

NOT AT ALL SOMEWHAT EASILY COMPLETELY EASILY

32. Was the information provided through different senses in the virtual environment (e.g., vision, hearing, touch) consistent?

|________|________|__________|_________|_________|_________|_________| NOT AT ALL SOMEWHAT CONSISTENT COMPLETELY CONSISTENT
