# Supplementary material for: Transfer of training—Virtual reality training with augmented multisensory cues improves user experience during training and task performance in the real world
Source: PLoS One. 2021 Mar 24;16(3):e0248225. doi: 10.1371/journal.pone.0248225 (PMC7990292; doi:10.1371/journal.pone.0248225)
Supplement: S3 Questionnaire — (PDF) [file pone.0248225.s003.pdf]

No_______________ Date_____________________

SIMULATOR SICKNESS QUESTIONNAIRE

Kennedy, Lane, Berbaum, & Lilienthal (1993)***

Instructions: Circle below how much each symptom below is affecting you right now.

1. General discomfort	None	Slight	Moderate	Severe
2. Fatigue	None	Slight	Moderate	Severe
3. Headache	None	Slight	Moderate	Severe
4. Eye strain	None	Slight	Moderate	Severe
5. Difficulty focusing	None	Slight	Moderate	Severe
6. Salivation increasing	None	Slight	Moderate	Severe
7. Sweating	None	Slight	Moderate	Severe
8. Nausea	None	Slight	Moderate	Severe
9. Difficulty concentrating	None	Slight	Moderate	Severe
10. « Fullness of the Head »	None	Slight	Moderate	Severe

11. Blurred vision	None	Slight	Moderate	Severe
12. Dizziness with eyes open	None	Slight	Moderate	Severe
13. Dizziness with eyes closed	None	Slight	Moderate	Severe
14. *Vertigo	None	Slight	Moderate	Severe
15. **Stomach awareness	None	Slight	Moderate	Severe
16. Burping	None	Slight	Moderate	Severe

* Vertigo is experienced as loss of orientation with respect to vertical upright.
** Stomach awareness is usually used to indicate a feeling of discomfort which is just short of nausea.
