# Supplementary material for: Transfer of training—Virtual reality training with augmented multisensory cues improves user experience during training and task performance in the real world
Source: PLoS One. 2021 Mar 24;16(3):e0248225. doi: 10.1371/journal.pone.0248225 (PMC7990292; doi:10.1371/journal.pone.0248225)
Supplement: S1 File — (PDF) [file pone.0248225.s004.pdf]

Figure 8.6

NASA Task Load Index

Hart and Staveland's NASA Task Load Index (TLX) method assesses work load on five 7-point scales. Increments of high, medium and low estimates for each point result in 21 gradations on the scales.

Name	Task	Date

Mental Demand — How mentally demanding was the task?

Very Low — Very High

Physical Demand — How physically demanding was the task?

Very Low — Very High

Temporal Demand — How hurried or rushed was the pace of the task?

Very Low — Very High

Performance — How successful were you in accomplishing what you were asked to do?

Perfect — Failure

Effort — How hard did you have to work to accomplish your level of performance?

Very Low — Very High

Frustration — How insecure, discouraged, irritated, stressed, and annoyed wereyou?

Very Low — Very High
