# Supplementary material for: Transfer of training—Virtual reality training with augmented multisensory cues improves user experience during training and task performance in the real world
Source: PLoS One. 2021 Mar 24;16(3):e0248225. doi: 10.1371/journal.pone.0248225 (PMC7990292; doi:10.1371/journal.pone.0248225)
Supplement: Attachment — Submitted filename: Response to reviewers.docx [file pone.0248225.s006.docx]

Dear Dr Fetch

Thank you – and the two reviewers – for taking the time to review the manuscript and to offer positive and constructive feedback.

We kept your comments from the decision letter and added our response in green to show how the comments were addressed.

------------------------------------------------------------------

Thank you for submitting your manuscript to PLOS ONE. After careful consideration, we feel that it has merit but does not fully meet PLOS ONE’s publication criteria as it currently stands. Therefore, we invite you to submit a revised version of the manuscript that addresses the points raised during the review process.

The reviewers were positive about the contribution and have only minor comments. In contrast, I have concerns about several of the claims being only weakly or not supported by statistical inference, and thus the manuscript requires several changes to soften or remove some of the claims, including but not limited to those referring to P values at or above 0.05. Specifically:

235, 284 - p=0.05 should not be described as significant, but I suspect these values are in fact slightly above or below 0.05, in which case please provide exact P values.

These have been amended as suggested.

237 - No effect on the last trial between the two groups? Doesn’t this at least partly undermine a principal claim of the paper? Even if other results support the hypothesis, it seems it would also predict a difference here, and thus not observing it deserves more than a perfunctory mention.

We disagree with the claim that *not* finding performance differences at the end of (the real) training undermines the principal claim. The key point to emphasize is that these trials are performance measures on the same (real) task *after* participants were trained in VR. The "study flow diagram" requested by reviewer 2 probably will help here.
Learning curves are exponential functions, their asymptotic behaviour means that we expect all participant groups to – eventually – arrive at comparable performance. In our experiment that is relatively fast, after 2 tyre changes we see little performance improvement in any of the three groups. The key argument we make is that VR training (and especially VR training with augmented cues) provides participants with a ‘head start’ when performing task in the real world. We do not expect to see significant performance differences towards the end of the training on the real task

236, 285, 310 - More importantly, best practices suggest it is misleading and inappropriate to refer to 0.1 > p > 0.05 as a “trend towards significance.” One way to appreciate this is to consider p values less than but close to 0.05, which by the same logic indicate a “trend toward non-significance.” Some recent views on this are here:

<https://www.bmj.com/content/348/bmj.g2215>

<https://academic.oup.com/bja/article/115/3/337/312358>

<https://www.ncbi.nlm.nih.gov/pmc/articles/PMC6440716/>

It is highly preferred to use phrases like “failed to reach significance” or simply “not significantly different” for such results, and any conclusions made based on them (including in Discussion) should be removed, or else described as a possibility that must be evaluated in future research.

The new sections have been added and the text has been updated accordingly.

Lastly, here is a list of minor fixes and suggestions on the writing. Note that PLOS ONE does not copyedit manuscripts, and so you’ll be stuck with any typos that make it past the review stage:

52 - add ‘but’ after comma

- done

122 - the ‘control’ group does not control for the possible placebo effect of having been trained. This is not a major problem but could be worth adding a caveat to the Discussion.

The text addressing this was added to the Discussion section.

133 - add ‘a’ or ‘the’ after “in” - done

235 - “that RO group” -> than the RO group - done

232, 236, 258 - “trail” -> trial – all replaced

275 - “a” -> the -done

275 -  “the bolts was not fully tighten” -> a bolt was not fully tightened – replaced with ‘when bolts were not fully tightened’

378 - “reduces” -> is reduced -done

397-399 - Please refer to which figure illustrates this result, and confirm its statistical significance per comments above.

The text in this section was amended as suggested.

447-448 - Same as above: indicate figure and confirm significance and not “trend towards”

The text in this section was amended as suggested

525 - “were” -> was - done

Please submit your revised manuscript by Feb 01 2021 11:59PM. If you will need more time than this to complete your revisions, please reply to this message or contact the journal office at [plosone@plos.org](mailto:plosone@plos.org). When you're ready to submit your revision, log on to <https://www.editorialmanager.com/pone/> and select the 'Submissions Needing Revision' folder to locate your manuscript file.

Please include the following items when submitting your revised manuscript:

- A rebuttal letter that responds to each point raised by the academic editor and reviewer(s). You should upload this letter as a separate file labeled 'Response to Reviewers'.
- A marked-up copy of your manuscript that highlights changes made to the original version. You should upload this as a separate file labeled 'Revised Manuscript with Track Changes'.
- An unmarked version of your revised paper without tracked changes. You should upload this as a separate file labeled 'Manuscript'.

If you would like to make changes to your financial disclosure, please include your updated statement in your cover letter. Guidelines for resubmitting your figure files are available below the reviewer comments at the end of this letter.

If applicable, we recommend that you deposit your laboratory protocols in protocols.io to enhance the reproducibility of your results. Protocols.io assigns your protocol its own identifier (DOI) so that it can be cited independently in the future. For instructions see: <http://journals.plos.org/plosone/s/submission-guidelines#loc-laboratory-protocols>

We look forward to receiving your revised manuscript.

5. Review Comments to the Author

Please use the space provided to explain your answers to the questions above. You may also include additional comments for the author, including concerns about dual publication, research ethics, or publication ethics. (Please upload your review as an attachment if it exceeds 20,000 characters)

Reviewer #1: The authors present a novel method to quantify the relative performance gains of students in virtual reality-enhanced learning environments. Specifically, the authors estimate differences between traditional training methods, traditional virtual reality training, and enhanced virtual reality environment.

The objective of the research as well as the methods are well explained. Also, the measurement instruments were well selected and led to meaningful results.

The discussion is well elaborated and accurately represents the research process. I consider that the manuscript does not need further adjustments.

Reviewer #2: Thank you very much for the opportunity to review this interesting manuscript which is an ongoing exploration of the addition of augmented multi sensory cues in the virtual environment and their effects on training and transfer of training into the real world environment. The authors conducted a study with 3 matched groups of participants assigned to "real world" tire change exercise, VR without augmented multi sensory cues training, or VR with augmented sensory cues training. I believe all participants performed 5 receptions of the "racing tire change" task with appropriate baseline assessments to confirm equivalence between groups.

Overall, the manuscript is well written and I feel can be accepted with minor revisions which I will outline below.

1. Introduction - well written and provides appropriate background for the reader to understand the material.
1.1. Ln 74 - I think you are missing "load" as part of "reduce cognitive". -done

2. Methods
2.1. Can the authors please provide a "study flow diagram" to explain graphically "baseline testing", number of repetitions performed in each group, testing performed after 1st attempt in each group, as well as clarify if and how many attempts participants in VR groups were allowed to make on the "real tire change" after training in the VR? I am still not clear on this point. Did participants in the VR groups perform 5 attempts in the VR and then 1 more attempt on "real tire change"?

The text to explain this further was added as well as the figure showing study flow diagram.

2.2. Please provide an explanation or definition for what "immersive tendencies" and "perceived sense of presence" means in the VR environment. This will help the readers familiarize themselves with these concepts.

We expanded the introductory section (L69-79) to introduce both concepts:

It is well understood that performance in VR training environments is directly affected by simulation fidelity [11–13]. Poor mechanical performance of simulated haptic feedback, for example, has been shown to result in negative training outcomes in surgical training [14]. Conversely, improved simulation fidelity not only captures the sensory cues and behaviours that learners encounter in real life better, but it also improves presence and immersion in VR environments [11–13,15–17] and reduce cognitive load [18–20]. ‘Immersion’ here stands for the objective level of sensory fidelity provided by a VR system, which is a direct measure of simulation fidelity. ‘Presence’ describes the subjective psychological response of a user experiencing a VR system as ‘being in the virtual world’ (see [7] for further discussion of these terms). For successful transfer of training from virtual into real environments, it therefore seems essential that the cues presented during the training in VR are representative of those encountered in reality.

2.3. Did you perform a power calculation to help you decide how many participants were required for each study group? If yes, please include this in the manuscript.

We did not perform a formal power analysis when planning the experiment: The main research question was whether VR training transfers to a real task, we would have had to assume an effect size. This experiments reported here, however, are a continuation of Cooper et al (2018) where we found highly significant differences in subjective and objective measures (effect sizes Cohen’s d=3.8, objective, and d=5.63, subjective) when comparing the two VR conditions chosen for the experiments reported in this study. A post-hoc power analysis (g-power, diff between independent means, alpha and beta =0.05, allocation ration=1) suggests that we would have ‘got away’ with just six participants (actual power 0.982) if our aim was to show the differences between augmented and ‘normal’ VR training. This is in line with data presented here [Cooper et al 2018 <https://doi.org/10.1371/journal.pone.0191846>]

2.4. Please move relevant descriptions of NASA TLX, ITQ, and SSQ questionnaires and their interpretation from Results section to Methods section.

This section has been amended.

3. Results
3.1. Ln 254 - 256 - can the authors please explain what this means? Does training in ATV VR is 80% as effective at training on "real life" model? Or, training in ATV VR is more effective than real life model. It is still confusing for me and I suspect will be for other readers.

The section was added to the text for better explanation.

The learning curve, also known as experience curve, represents the improved efficiency or performance obtained from repeating an operation of a specific task by a worker; i.e. the time required to perform a task declines at a decreasing rate as experience with the task increases [33].

To enable a direct comparison of the data the three curves were fitted simultaneously. Parameters A1, x¬1 and y0 were shared across the three data sets under the assumption that, since each of the three (matched) groups performed the same task, we assume initial and final performance as well as learning rate to be the same for all three groups. The x-offset (x0), in contrast, was fitted for each individual to quantify the equivalent training time (number of trial runs) that was gained by participating in the VR training exercise preceding the real tyre changes. The difference in x0¬ between the ‘real only’ (RO) condition and the two VR training conditions therefore represents the ‘real training time saved’ by taking part in the initial VR training.

3.2. Ln 265 - similar to my comment above, please explain this a bit more. Do you expect individuals to achieve the same "level of skill" or "outcome" by training 2/3 of the total time in ATV VR as compared to real life? Is it more effective?

The section has been added to the text to provide a better explanation.

To estimate the shift parameter (x0), a bootstrapping analysis with 10000 resamples (with replacement) was carried out to estimate the mean x-offset (x0) and 95% confidence intervals for each of the three conditions. The mean x-offset ((x0) ̅) for the RO group was 1.10 (CI 0.51 - 1.63), for the NC group (x_0 ) ̅ = 0.554 (CI -0.0423 - 1.171) while for the ATV group (x_0 ) ̅ = 0.1735 (CI -0.3903 - 0.6853). The probability that the observed mean x-offset values were drawn from the RO data are p=0.0012 (ATV) and p=0.0329 (NC) respectively. The relative differences in the x0 parameter mean that conventional VR training (NC) is equivalent to 0.37 ‘training sessions’ on a real tyre while the augmented (ATV) VR training provides gains that are equivalent to 0.93 real tyre changes (Fig 3).

4. Discussion
4.1. Ln 388-389 - can you please point me to the data to support the statement that realism of the VR training is decreased? I was not able to find this in the manuscript.

A more detailed explanation is presented in the text. Line 107-119 also explain this.

4.2. Ln 393 - 394 - can you please point me to the results that support the statement that both VR groups performed better on real life task than RO group? This goes back to my comment previously about not being able to understand if VR group participants actually performed greater number of total repetitions (VR + real life) than RO participants. If yes, this is a possible explanation for this finding that should be discussed.

The text has been added as well as study flow diagram.

4.3. Ln 453 - can you please indicate where "surface fidelity" was measured and how?

Neither ‘surface fidelity’, nor ‘informational fidelity’, the two terms we use in the context, were measured. However, there is a well-rehearsed argument that an “exact measure of realism is not feasible at this time, and is considered by some to be “a goal which can never be accomplished” (Roza et al., 2001).” (from Liu et al., Liu, D., Macchiarella, N. D., & Vincenzi, D. A. (2008). Simulation fidelity. Human factors in simulation and training, 61-73.)

To address this comment we expanded our text to clearly define the terms we use from the outset. The key point we make is that we deliberately introduce signals, which cannot occur in reality, the colour-change when a tyre is correctly seated, for examples. These changes, by definition, reduce ‘surface fidelity’.

4.4. Consider including a limitations section as one is not found in the manuscript.

This section has been added.

4.5 Ln - 515 - can you please explain why addition of cues results in reduction of simulation fidelity? I would think that adding more "sounds", "haptics", etc. would increase fidelity as long as there is task alignment.

A section explaining this has been added to the introduction because it is a key consideration for the experiments.

Boris Zevin MD, PhD

6. PLOS authors have the option to publish the peer review history of their article ([what does this mean?](https://journals.plos.org/plosone/s/editorial-and-peer-review-process#loc-peer-review-history)). If published, this will include your full peer review and any attached files.


If you choose “no”, your identity will remain anonymous but your review may still be made public.


**Do you want your identity to be public for this peer review?** For information about this choice, including consent withdrawal, please see our [Privacy Policy](https://www.plos.org/privacy-policy).

Reviewer #1: **Yes:** Juan Garzón

Reviewer #2: **Yes:** Boris Zevin MD, PhD

[NOTE: If reviewer comments were submitted as an attachment file, they will be attached to this email and accessible via the submission site. Please log into your account, locate the manuscript record, and check for the action link "View Attachments". If this link does not appear, there are no attachment files.]

While revising your submission, please upload your figure files to the Preflight Analysis and Conversion Engine (PACE) digital diagnostic tool, <https://pacev2.apexcovantage.com/>. PACE helps ensure that figures meet PLOS requirements. To use PACE, you must first register as a user. Registration is free. Then, login and navigate to the UPLOAD tab, where you will find detailed instructions on how to use the tool. If you encounter any issues or have any questions when using PACE, please email PLOS at [figures@plos.org](mailto:figures@plos.org). Please note that Supporting Information files do not need this step.
